# Long-term psychosocial sequelae of Ebola virus disease among survivors compared with contacts following the 2013–2016 epidemic in Liberia

**DOI:** 10.1371/journal.pgph.0005145

**Published:** 2026-01-27

**Authors:** Moses Badio, Meekie Glayweon, Collin Van Ryn, Barthalomew Wilson, Joseph B. Cooper, J. Soka Moses, Tamba Fayiah, Kumblytee Johnson, Dehkontee Gayedyu-Dennis, Smit D. Chitre, Eugene T. Richardson, Cavan Reilly, Sheri D. Weiser, George W. Rutherford, Mosoka P. Fallah, J. Daniel Kelly

**Affiliations:** 1 Partnership for Research on Vaccines and Infectious Diseases in Liberia, Monrovia, Liberia; 2 Department of Epidemiology and Biostatistics, University of California, San Francisco, California, United States of America; 3 Division of Biostatistics and Data Health Science, School of Public Health, University of Minnesota, Minneapolis, Minnesota, United States of America; 4 Department of Global Health and Social Medicine, Harvard Medical School, Boston, Massachusetts, United States of America; 5 Department of Medicine, Brigham and Women’s Hospital, Boston, Massachusetts, United States of America; 6 Department of Medicine, University of California, San Francisco, California, United States of America; 7 Institute for Global Health Sciences, University of California, San Francisco, California, United States of America; 8 Achille Mario Dogliotti College of Medicine, University of Liberia, Monrovia, Liberia; 9 Francis I. Proctor Foundation, University of California, San Francisco, California, United States of America; PLOS: Public Library of Science, UNITED STATES OF AMERICA

## Abstract

Long-term psychosocial sequelae of Ebola virus disease (EVD) survivors are poorly understood. We conducted a cross-sectional study of a cohort of EVD survivors and their uninfected contacts in Liberia. Beginning in November 2019, we consecutively sampled eligible participants until the end of the parent study follow up. Enrolled participants completed a questionnaire administered by trained study staff. Outcome measurements were symptoms of depression (PHQ-8) and anxiety (GAD-7) as well as experiences of trauma (PC-PTSD-5), food insecurity (HFIAS), and social support (Duke-UNC SSQ)*.* Excess prevalence was defined as the difference in prevalence between survivors and contacts. We performed adjusted analyses with logistic regression models and restricted to the survivor population for assessment of risk factors. Our analysis cohort included 1,144 participants among whom 363 were Ebola survivors and 781 were contacts. Participants were sampled a median of 55 months (IQR: 51, 60) after enrollment in the parent study. Excess prevalence was as follows: 7.9% had severe food insecurity by HFIAS (18.5% EVD survivors vs. 10.6% contacts), 6.2% had any anxiety symptoms by GAD-7 (11.6% EVD survivors vs. 5.4% contacts), 3.7% had any depression symptoms by PHQ-8 (8.3% EVD survivors vs. 4.6% contacts), and 3.2% had probable PTSD by PC-PTSD-5 (4.5% EVD survivors vs. 1.3% contacts). Levels of support were similar between survivors and contacts. EVD survivors had a higher adjusted odds of scoring in a more severe category on the GAD-7 scale (adjusted odds ratio [AOR]: 3.60; 95% CI: 1.42, 9.16), PC-PTSD-5 scale (AOR: 3.26; 95% CI: 1.40, 7.58), and HFIAS scale (AOR: 1.84; 95% CI: 1.29, 2.61). Age, history of post-acute symptoms, and non-Ebola related trauma were risk factors for multiple psychosocial outcomes among survivors. We found significant but modest evidence of psychosocial sequelae among EVD survivors compared with contacts between four to five years after the acute illness.

## Introduction

Following Ebola virus disease (EVD), survivors can experience a variety of post-acute sequelae [1–8], including psychosocial issues [9–12]. The 2013–2016 EVD epidemic in West Africa was unprecedented in scale, surpassing all previous EVD outbreaks in cases, deaths, and survivors. The large quantity of EVD survivors allowed for a thorough characterization of their psychosocial distress in the post-acute period, which encompassed stigma from fear of contagion [13–16], depression from loss of life [17–20], decreased wellbeing [21], food insecurity from work-related disability [22], addiction to substances [12,23], and other psychosocial consequences. These and other post-acute sequelae galvanized the Ebola response community to create comprehensive survivor programs [24–27], but after a few years, most of these programs stopped [22].

As reported in a systematic review and meta-analysis by Cénat and colleagues, the prevalence of mental health disorders in EVD-affected populations, inclusive of survivors, showed consistently high levels of psychological distress, but almost all of the included studies concluded their observational period within two years of the EVD outbreak ending [28]. Similarly, a systematic review by James and colleagues that found various forms of psychosocial distress among EVD survivors predominantly included studies that evaluated survivors within one year of discharge; the authors themselves highlight a need for “well-designed longitudinal studies” assessing the long-term psychosocial issues experienced by EVD survivors [12].

Although the psychosocial sequelae have been well described over the first couple of years following the 2013–2016 EVD epidemic in West Africa, as well as other EVD outbreaks [29,30], there is limited but compelling evidence of long-term psychosocial sequelae in EVD survivor populations [31–33]. A > 20-year follow-up study of EVD survivors and contacts found that symptoms of depression and anxiety were more prevalent among survivors than contacts [10]. This study was small in size and generated the hypothesis that there may be long-term psychological sequelae in other EVD outbreaks such as the 2013–2016 one in West Africa.

In addition to the EVD outbreak, many Liberian people have experienced more than a decade of civil war, extreme poverty, and low life expectancy, so there numerous biosocial forces that intersect to determine wellbeing [34,35]. As such, to adequately understand the psychosocial distress attributable to having survived EVD, an uninfected group was needed to serve as a comparator/control. The PREVAIL Ebola Natural History Study (PREVAIL III) enrolled both survivors and uninfected comparators (seronegative contacts) and followed them up to 5 years. Towards the end of the follow-up period, a sub-study was created to assess the prevalence of psychosocial findings in these participants. Our hypothesis was that >3 years post-outbreak, psychosocial findings would be more prevalent among the EVD survivor group than the uninfected, comparator group.

## Methods

### Ethics approval

The study protocol was approved by the National Research Ethics Board of Liberia, the University of California, San Francisco Institutional Review Board, and the National Institute of Allergy and Infectious Diseases Institutional Review Board (NIAID IRB) at the United States National Institutes of Health. Written informed consent was obtained from all participants. Assent was obtained from minors.

### Study design

We conducted a cross-sectional study on psychosocial findings among Ebola survivors and seronegative contacts enrolled in a longitudinal observational cohort study in Liberia called the PREVAIL Natural History Study (PREVAIL III). This parent study was established during the Ebola outbreak as a collaboration between NIAID/NIH and the Liberian Ministry of Health. The study was conducted at three sites (JFK medical center, Duport Road Health Center, and CH Rennie Hospital) in Liberia (Montserrado and Margibi counties).

### Study participants

EVD survivors of all ages listed on the Ministry of Health registry were enrolled. Each survivor identified up to 9 close contacts who were then recruited into the study from 06/01/2015 until 06/30/2017. Close contacts with no history of EVD were invited to enroll as controls. These controls lived in the same community as the survivor, and in many scenarios, the same household. More details about the parent study can be found elsewhere [36]. In this sub-study of psychosocial sequelae, we excluded participants <12 years because of concerns about understanding the questionnaire and excluded contacts with seropositive results because of potential misclassification bias (others have corroborated the finding in our cohort that most seropositive contacts [missed EVD cases] are asymptomatic/pauci-symptomatic [5,37–40]). From 11/01/2019 until 12/31/2021, we selected eligible participants being followed within in the parent study, using a non-probabilistic, consecutive sampling strategy. Enrolled participants completed a paper-based questionnaire administered orally in English (or Liberian English) by trained study staff, including medical monitors and psychosocial counselors. Study staff entered collected data into an electronic data capture system and scanned these forms for data review at the data coordinating center.

### Measurements

Outcome measurements were the eight-item Patient Health Questionnaire depression scale (PHQ-8); seven-item General Anxiety Disorder-7 (GAD-7); five-item primary care, post-traumatic stress disorder (PC-PTSD-5) screener; nine-item household food insecurity and access survey (HFIAS); and the Duke-UNC functional social support questionnaire (SSQ). These measures were selected because they have been previously validated and used in Liberia and other West African populations [12,28]. These surveys were piloted by the staff. The surveys did not require adaptation prior to study start because English is the national language of Liberia. However, potential discrepancies between the local English dialect and validated English text existed, so a translated version of these particular words were jointly administered to optimize understanding.

These survey instruments, respectively, can be considered surrogate measurements for symptoms of depression and anxiety as well as for experiences of trauma, food insecurity, and social support*.* The PHQ-8 is established as a diagnostic severity measure for depressive disorders. The GAD-7 is an efficient tool for screening and severity measuring of generalized anxiety disorder. PC-PTSD-5 is designed to identify individuals with probable PTSD in primary care settings. HFIAS captures households’ behavioral and psychological manifestations of insecure food access [41]. SSQ is a tool for measuring multiple dimensions of social support.

The following covariates were included from the questionnaires: age, sex, education, current and pregnancy in the last 6 months, HIV status, hospitalization since acute EVD, and history of any non-Ebola related traumatic event. The definition of any non-Ebola related traumatic event was determined in the following manner: 1. Eliciting the presence of stress related to traumatic events; 2. If stress was present, the event(s) were identified from structured and free-response options that were related to Ebola (e.g., ETU-related, event in community related to Ebola, other Ebola event) and/or non-Ebola (e.g., accident, natural disaster, conflict-related, violence); and 3. If any non-Ebola related traumatic events were selected, the participant was determined to have a history of non-Ebola related traumatic event. This and the other covariates were included in the risk factor analysis because of their potential association with the psychosocial outcome measures among EVD survivors based on the literature and expert review.

In addition, a series of symptoms or exam abnormalities had been identified to be of significantly higher prevalence among survivors than uninfected comparators (seronegative contacts), including urinary frequency, headache, fatigue, muscle pain, joint pain, and memory loss, uveitis, as well as neurologic, chest, musculoskeletal and abdominal findings. These symptoms or exam abnormalities were reported by a participant at or after enrollment and were used to create two covariates: history of any symptom and history of any exam abnormality.

### Statistical analysis

We conducted a descriptive analysis of the cohort stratified by survivors and seronegative contacts. The psychosocial outcomes of GAD-7 (scale, 0–21; presence of any anxiety symptoms defined as a score of 5 or greater) and PHQ-8 (scale, 0–24; presence of any depressive symptoms defined as a score of 5 or greater) were described as categorial variables. PC-PTSD-5 (score 10 or more, binary), HFIAS (severe or not severe category, binary) and Duke-UNC Functional SSQ (response of 3 or less, binary) were dichotomized to characterize probable PTSD, severe food insecurity, and average social support, respectively. We described excess prevalence of psychosocial findings among EVD survivors by subtracting the prevalence of a finding among survivors by the prevalence among contacts.

Comparisons of psychosocial findings among EVD survivors and close contacts were assessed for differences using regression models. For categorical outcomes, a multivariable proportion-odds logistic regression model was developed to estimate the odds ratios for being in a more severe category according to the GAD-7 and PHQ-8 while adjusting for age, sex, and education level and clustering for the relationships between survivors and contacts. For binary outcomes, we employed multivariable logistic regression models, using the same adjustment set and clustering variable. We then restricted the sample to EVD survivors and used regression models to assess the association between risk factors (age, sex, education, pregnancy status, HIV status, hospitalization since EVD, history of symptoms, history of exam findings, history of uveitis, and history of non-Ebola related trauma) and each psychosocial outcome adjusting for age, sex and education.

Our threshold for a statistically significant finding was either lower limit of confidence interval above the null value of 1 or p-value less than 0.05. Analyses were performed using RStudio (R Project for Statistical Computing, Vienna, Austria; version 3.2.3).

### Inclusivity in global research

Additional information regarding the ethical, cultural, and scientific considerations specific to inclusivity in global research is included in the Supporting Information (S1 File).

## Results

The parent study included 1144 survivors and 2784 close contacts, totaling 3928 participants. Of these, 363 (32%) of enrolled survivors and 781 (28%) of enrolled close contacts, or 29% of the total enrollment, are included in this analysis. The median time since enrollment in the parent study was 55 months (IQR: 51, 60), and participants were enrolled approximately 12 months after discharge from an Ebola treatment unit. The majority of participants were female (55.0%) while less than quarter were age 50 or more (14.2%), had no formal education (19.0%), and had history of any non-Ebola related traumatic event (14.3%). A smaller proportion was pregnant at the time of interview (5.4%) or tested positive for HIV (2.4%) (Table 1).

**Table 1:** Characteristics of EVD survivors and seronegative contacts between 30 and 63 months after enrollment (median (Q1, Q3): 55 (51, 60)). Counts and percentages (N (%)) are shown.

	Total (n = 1144)	EVD survivors (n = 363)	Seronegative contacts (n = 781)
Female	629 (55)	205 (56.5)	424 (54.3)
Age 12–19	197 (17.2)	41 (11.3)	156 (20)
Age 20–29	327 (28.6)	72 (19.8)	255 (32.7)
Age 30–39	262 (22.9)	102 (28.1)	160 (20.5)
Age 40–49	195 (17)	79 (21.8)	116 (14.9)
Age 50+	163 (14.2)	69 (19)	94 (12)
Education (No formal education)	214 (18.7)	74 (20.4)	140 (17.9)
Education (Primary, junior high, vocational)	554 (48.4)	154 (42.4)	400 (51.2)
Education (high school or beyond)	376 (32.9)	135 (37.2)	241 (30.9)
Current pregnancy (% of females)	33 (5.4)	11 (5.6)	22 (5.3)
Pregnant in the last 6 months	32 (6.2)	11 (7.3)	21 (5.8)
HIV positive	27 (2.4)	7 (2)	20 (2.6)
Hospitalized since acute EVD	15 (4.1)	15 (4.1)	–
History of any symptom differentially endorsed by EVD survivors at or after enrollment (urinary frequency, headache, fatigue, muscle pain, joint pain, memory loss)	871 (76.2)	330 (90.9)	541 (69.4)
History of any exam abnormality differentially endorsed by EVD survivors at or after enrollment (neuro- logic, chest, musculoskeletal, and abdominal findings)	552 (48.7)	219 (61.3)	333 (42.9)
History of uveitis at or after enrollment	187 (31.9)	138 (42.9)	49 (18.5)
History of any non-Ebola related traumatic event	164 (14.3)	50 (13.8)	114 (14.6)

Variable (n missing): Sex (0), Education (0), Pregnancy (19), Pregnant in the last 6 months (116), HIV status (27), Hospitalization since EVD (0), Symptom history (1), Exam finding history (10), Uveitis history (557), Non-Ebola related traumatic event history (0).

### Description of psychological findings

The overall cohort, roughly two-thirds of which was comprised of seronegative contacts, reported all psychosocial outcomes with a relatively low prevalence. Any occurrence of severe food insecurity was the most prevalent finding (13.1%). A low proportion of participants reported GAD-7 assessed the presence of any anxiety symptoms, defined as a score of 5 or greater (7.3%); PHQ-8 assessed the presence of any depression symptoms, defined as a score of 5 or greater (5.6%); and had a positive PC-PTSD-5 screener (2.3%).

Excess prevalence among survivors, defined as the difference in prevalence between survivors and contacts, was as follows (in order of most to least common): 7.9% had severe food insecurity by HFIAS (18.5% EVD survivors vs. 10.6% contacts), 6.2% had any anxiety symptoms by GAD-7 (11.6% EVD survivors vs. 5.4% contacts), 3.7% had any depression symptoms by PHQ-8 (8.3% EVD survivors vs. 4.6% contacts), and 3.2% had probable PTSD by PC-PTSD-5 (4.5% EVD survivors vs. 1.3% contacts). Almost all participants (95.7%) reported higher than average levels of social support, and levels of support were similar between survivors and contacts.

### Associations with psychosocial findings among EVD survivors

While the point estimates were higher in survivors than contacts, the differences were statistically significant for the scales associated with symptoms of anxiety, probable PTSD and severe food insecurity. EVD survivors had a higher adjusted odds of scoring in a more severe category on the GAD-7 scale (adjusted odds ratios [AOR]: 3.60; 95% CI: 1.42, 9.16), on the PC-PTSD-5 scale (AOR: 3.26; 95% CI: 1.40, 7.58), and on the HFIAS scale (AOR: 1.84; 95% CI: 1.29, 2.61). Scoring in a more severe category on the PHQ-8 scale and on the SSQ scale were not associated with survivor status (Table 2).

**Table 2:** Description and comparisons of psychosocial risk factors and quality of life among EVD survivors and seronegative contacts. Counts and percentages (N (%)) are shown. Odds ratios are adjusted for age, sex, and education level. Odds ratios for the GAD-7 and PHQ-8 refer to the odds of being in a more severe category. All models were additionally adjusted for relationships between survivors and contacts.

	Total (n = 1144)	EVD survivors (n = 363)	Seronegative contacts (n = 781)	Odds Ratio (95% CI)
Symptoms of anxiety (GAD-7 total score)				
Minimal (0–4)	1060 (92.7)	321 (88.4)	739 (94.6)	
Mild (5–9)	66 (5.8)	33 (9.1)	33 (4.2)	
Moderate (10–14)	7 (0.6)	3 (0.8)	4 (0.5)	
Severe (15–21)	11 (1)	6 (1.7)	5 (0.6)	3.6 (1.42, 9.16)
Symptoms of depression (PHQ-8 total score)				
None (0–4)	1078 (94.2)	333 (91.7)	745 (95.4)	
Mild (5–9)	46 (4)	21 (5.8)	25 (3.2)	
Moderate (10–14)	12 (1)	7 (1.9)	5 (0.6)	
Moderately severe (15–19)	5 (0.4)	0 (0)	5 (0.6)	
Severe (20–24)	3 (0.3)	2 (0.6)	1 (0.1)	1.52 (0.63, 3.68)
PC-PTSD-5 screener Probable PTSD	24 (2.3)	15 (4.5)	9 (1.3)	3.26 (1.4, 7.58)
Household food insecurity (HFIAS score) Severe (any occurrence)	150 (13.1)	67 (18.5)	83 (10.6)	1.84 (1.29, 2.61)
Social support (Duke-UNC Func- tional SSQ)				
Average response *<*3 (“Almost as much...”)	49 (4.3)	17 (4.7)	32 (4.1)	1.03 (0.56, 1.91)

Variable (n missing): GAD-7 (0), PHQ-8 (0), PC-PTSD-5 (116), HFIAS (0), SSQ (0)

### Risk factors for psychosocial sequelae

Risk factors for psychosocial sequelae among EVD survivors were consistent across multiple outcomes. The age group of 20–39 was a risk factor for each psychosocial outcome (p < 0.05). Symptoms of anxiety, as evaluated by the GAD-7, and depression, as evaluated by the PHQ-8 showed the strongest associations with a history of post-acute symptoms (GAD-7: p = 0.037; PHQ-8: p = 0.051) and non-Ebola related trauma (GAD-7: p < 0.001; PHQ-8: p < 0.001). No significant associations were found with sex, education, pregnancy, HIV status, hospitalization since Ebola, or specific medical conditions such as uveitis (Table 3).

**Table 3:** Psychosocial risk factors among EVD survivors by selected demographics factors. P-values are adjusted for sex, age, and education.

	GAD-7 score		PHQ-8 score		Probable PTSD (PC-PTSD-5)		Food Insecure (HFIAS)		Modified Duke-UNC Functional SSQ score	
	Mean*±*SD	*p*-value	Mean*±*SD	*p*-value	N (%)	*p*-value	N (%)	*p*-value	Mean*±*SD	*p*-value
Male	1.54*±*2.81		1.15*±*2.31		6 (4.2)		28 (17.7)		29.94*±*3.04	
Female	1.54*±*3.18	0.796	1.37*±*3.15	0.342	9 (4.7)	0.508	39 (19)	0.924	30.24*±*3.19	0.84
Age 12–19	0.22*±*0.57		0.1*±*0.49		0 (0)		2 (4.9)		31.22*±*1.8	
Age 20–29	1.49*±*3.7		1.35*±*3.75		2 (3)		8 (11.1)		30.74*±*3.26	
Age 30–39	2.62*±*3.89		1.9*±*3.29		8 (8.7)		25 (24.5)		29.69*±*3.56	
Age 40–49	1.2*±*1.98		0.9*±*1.67		1 (1.4)		21 (26.6)		30.13*±*2.6	
Age 50+	1.16*±*2.03	0.005	1.41*±*2.56	0.031	4 (6.3)	0.039	11 (15.9)	0.002	29.42*±*3.22	0.032
Education (No formal education)	1.43*±*3.35		1.18*±*3.12		2 (2.8)		18 (24.3)		30.19*±*3.96	
Education (Primary, jr. high, vocational)	0.99*±*2.21		1.02*±*2.2		6 (4.2)		27 (17.5)		30.51*±*2.52	
Education (High school or beyond)	2.22*±*3.49	0.129	1.62*±*3.22	0.529	7 (5.8)	0.422	22 (16.3)	0.193	29.62*±*3.18	0.267
Not currently pregnant	1.57*±*3.4		1.32*±*3.35		7 (5.3)		25 (17.7)		30.38*±*3.42	
Currently pregnant	0.82*±*2.09	0.94	0.82*±*1.47	0.973	0 (0)	0.994	1 (9.1)	0.492	30.91*±*1.51	0.769
Not pregnant in the last 6 months	1.62*±*3.44		1.38*±*3.36		7 (5.4)		26 (18.6)		30.36*±*3.43	
Pregnant in the last 6 months	0.36*±*0.81	0.284	0.18*±*0.6	0.295	0 (0)	0.994	0 (0)	0.989	30.91*±*1.38	0.606
HIV negative	1.48*±*2.97		1.23*±*2.75		13 (4.1)		63 (18.4)		30.16*±*3.07	
HIV positive	1.43*±*1.62	0.843	0.43*±*1.13	0.328	0 (0)	0.993	0 (0)	0.987	31.57*±*1.13	0.148
Not hospitalized since EVD	1.55*±*2.99		1.29*±*2.81		15 (4.7)		66 (19)		30.06*±*3.17	
Hospitalized since EVD	1.33*±*3.87	0.981	0.93*±*3.08	0.796	0 (0)	0.989	1 (6.7)	0.267	31.27*±*1.16	0.288
No history of symptoms	0.45*±*1.09		0.21*±*0.78		0 (0)		6 (18.2)		31.06*±*1.98	
History of symptoms	1.65*±*3.13	0.037	1.38*±*2.92	0.051	15 (4.9)	0.989	61 (18.5)	0.901	30.02*±*3.2	0.149
No history of exam findings	1.19*±*2.59		0.95*±*2.12		4 (3.1)		28 (20.3)		30.46*±*2.4	
History of exam findings	1.78*±*3.29	0.076	1.52*±*3.19	0.15	11 (5.5)	0.49	39 (17.8)	0.358	29.9*±*3.51	0.131
No history of uveitis	1.57*±*3.08		1.27*±*2.77		8 (4.7)		33 (17.9)		30.09*±*3.41	
History of uveitis	1.62*±*2.99	0.969	1.35*±*2.89	0.91	5 (4)	0.703	30 (21.7)	0.614	29.88*±*2.93	0.893
No history of non-Ebola related trauma	1.29*±*2.49		1.03*±*2.21		14 (4.5)		53 (16.9)		30.28*±*2.92	
History of non-Ebola related trauma	3.1*±*5.03	*<* 0.001	2.82*±*4.95	*<* 0.001	1 (4.5)	0.904	14 (28)	0.094	29.04*±*4.06	0.035

## Discussion

In this cross-sectional study of a large Liberian cohort, we found significant but modest evidence of psychosocial sequelae among EVD survivors after comparing findings with controls between four to five years after acute illness. These findings extend an existing body of evidence that psychosocial sequelae can occur in the first few years after acute illness and continue for up to several decades [10]. Consistent with the literature [28,35], multiple psychosocial assessments of EVD survivors indicated that they may be experiencing a higher burden of mental illness including symptoms of anxiety, probable PTSD, and food insecurity [19,28]. Implications of these findings suggest that there may be an ongoing need for psychosocial counseling in these different areas.

In our study, psychosocial findings were primarily mild in severity, which may represent either decreasing stressors or adequate coping mechanisms. For example, only 1.7% of EVD survivors reported severe symptoms of anxiety, as measured by the GAD-7**.** In the immediate aftermath of the epidemic, there were some EVD reports of a higher burden of more severe mental illness and psychological distress [28]. Even with the identification of mild psychosocial findings, we were still able to highlight multiple risk factors (age, non-Ebola related trauma) which can be used more immediately after surviving EVD to screen and engage individuals who may benefit from psychosocial counseling**.** These findings are consistent with other EVD studies assessing risk factors for stigma and other psychosocial outcomes [13,14]. Given the significant but modest evidence of psychosocial sequelae among EVD survivors, use of these risk factors in screening tools may be considered as part of an efficient population-based strategy to identify the small population of survivors with long-term mental health impacts of EVD during primary care encounters.

This study has several limitations. First, we enrolled participants several years after their participation in PREVAIL III, so we lack data on the severity of their psychosocial distress earlier in the post-acute period. Second, participants were aware of their survivorship status which could introduce measurement bias. However, as these participants had been involved in PREVAIL for many years and did not receive direct benefits for their research responses, and because assessments were conducted by trained psychosocial counselors and medical monitors who confirmed the findings, measurement bias may be limited. Third, the measurement tools used in the study serve as screening rather than diagnostic tools, so findings need to be interpreted with caution. Fourth, participants may have been inclined to underreport psychosocial issues due to sociocultural stigma or social desirability bias. Fifth, the initial questionnaires were designed without this study in mind, leaving potentially unmeasured confounders, such as severity of acute illness or levels of instrumental social support received as part of survivor programs; Richardson and colleagues showed that different levels of short-term social protection during the vulnerable period post-discharge from an Ebola Treatment Unit had significant impacts on survivor wellbeing and food security years after the outbreak in Sierra Leone [24]. Sixth, since we excluded individuals under the age of 12 due to concerns about their ability to comprehensively understand the questionnaire, we do not have data on the prevalence and characteristics of psychosocial sequelae in younger EVD survivors. Given the psychosocial outcomes may have been different among older survivors, excluding younger survivors from our study could potentially have influenced the prevalence reported in the EVD survivor population. Seventh, the PHQ-8 and similar screening tools, developed in affluent North Atlantic contexts, may face limitations when applied to settings with potentially high baseline levels of psychosocial distress due to historical and structural factors, including colonialism, neocolonialism, and corporate extractivism. Distress from these historical and structural factors was not measured, so some degree of distress may be mis-attributed to EVD.

In summary, in a country like Liberia that has experienced war, extractivism, chronic underdevelopment, and other forms of structural violence embodied as epidemic disease [42]—in addition to the Ebola crisis [34,35,43–46]—there is a great need for psychosocial services especially among those surviving infectious disease with long-term effects. In the wake of the Ebola epidemic, Liberia was home to only one registered psychiatrist; no psychologists; and no nurses, social workers, or physician’s assistants with mental health training [47]. As such, Liberia did not have a formally established mental health system, according to the World Health Organization [48].

Since then, the World Health Organization, several NGOs, and the Liberian Ministry of Health have implemented programs to train hundreds of clinicians and establish wellness units with community resource centers to strengthen mental health activities [49]. While the success of these programs remains unclear, it is imperative that they support the unaddressed and ongoing need for psychosocial services among EVD survivors during this and future outbreaks. Our findings exhibit that Ebola survivors had a higher, albeit modest, prevalence of psychosocial sequelae, which may benefit from tailored community and individual support.

We want to thank the EVD survivors and contacts for their participation and the PREVAIL team for making this study possible. We appreciate the input and support of H. Clifford Lane and Jeffrey N. Martin.

## Supporting information

S1 File Inclusivity in global research. (DOCX)
